# Impact of a non-return-to-work prognostic model (WORRK) on allocation to rehabilitation clinical pathways: A single centre parallel group randomised trial

**DOI:** 10.1371/journal.pone.0201687

**Published:** 2018-08-02

**Authors:** Chantal Plomb-Holmes, Roger Hilfiker, Bertrand Leger, François Luthi

**Affiliations:** 1 Department for Musculoskeletal Rehabilitation, Clinique Romande de Réadaptation suvacare, Sion, Switzerland; 2 Institute for Research in Rehabilitation, Clinique Romande de Réadaptation suvacare, Sion, Switzerland; 3 School of Health Sciences, University of Applied Sciences and Arts Western Switzerland Valais (HES-SO Valais-Wallis), Sion, Switzerland; 4 Department of Physical Medicine and Rehabilitation, Orthopaedic Hospital, Lausanne University Hospital, Lausanne, Switzerland; University of Birmingham, UNITED KINGDOM

## Abstract

**Introduction:**

Stratified medicine might allow improvement of patient outcomes while keeping costs stable or even diminishing them. Our objective was to measure if a prediction model, developed to predict non-return to work (nRTW) after orthopaedic trauma, improves the allocation to various vocational pathways for use in clinical practice.

**Material and methods:**

Randomised-controlled trial on vocational inpatients after orthopaedic trauma (n = 280). In the intervention group, nRTW risk (estimated using the WORRK tool) was given to the clinician team before allocation of vocational pathways, while in the control group it was not. Three pathways were available: simple, coaching and evaluation (EP). Accompanying indications for interpretation of the nRTW risk were given. The primary outcome was the proportion of patients allocated to the EP. The secondary outcome was patients’ and clinicians’ satisfaction.

**Results:**

450 patients were assessed for eligibility, 280 included, 139 randomized to the control group (mean age 42.3years) and 141 to the intervention group (43.2years). The two groups had a similar risk profile. The patients in the intervention group were more often referred to the EP compared to the control group, but not statistically significantly more (risk ratio 1.31 [95% CI 0.70–2.46]). The number needed to treat was 30. When considering patients transferred to different pathways during rehabilitation, more patients from the intervention group were transferred to the EP over the course of the rehabilitation, increasing the risk ratio to 1.57 [95% CI 0.89 to 2.74].

**Discussion:**

The knowledge of the risk of nRTW has an influence, that is not however statistically significant and is without clinical importance as previously defined by our own power calculations (based on a 15% increase in referral to EP in the intervention group compared to the control group), on clinical decision making with regards to the allocation of patients to different physical and vocational rehabilitation programs after orthopaedic trauma. This influence is less than what was expected, possibly due to insufficient directive guidelines accompanying the WORRK model, or because clinicians associate less hours of therapy (as with certain rehabilitation programs) to disadvantaging the patient. These findings do, however, support the multi-factorial aspect of clinician decision-making.

## Introduction

Work disability, defined as cessation of work due to illness, injury or any other medical cause, constitutes a vast economic and social burden, with more than 40 million disabled people of working age in the European Union, largely due to psychiatric illnesses and musculoskeletal disorders, and in particular, non-fatal, minor to moderate professional and non-professional orthopaedic traumas [1–5]. In addition to the financial load, work disability and more specifically orthopaedic trauma has a variety of consequences on patients, often leading to substantial psychosocial strain, affecting quality of life, reducing physical activity, causing chronic pain and leading to prolonged absence from work, a factor which can again have a negative effect on health (physical and psychological) as well as social integration [5–8]. Orthopaedic and vocational rehabilitation plays an important role not only in the costs incurred by musculoskeletal trauma, but also in determining patient outcomes [9, 10]. There is therefore room for development in the functioning of rehabilitation centres, particularly in their attribution of resources, in order to alleviate not only economic, but also patient-related physical, psychological and social strains.

Research is currently being directed towards what is called stratified medicine: treatment decisions are made according to the biological or risk characteristics of a patient, and therefore their likely response to the treatment in question [11]. This ideally allows for the improvement of patient outcomes while keeping costs stable, if not diminishing them. In order to attain this type of practice, prognostic research must follow a certain framework. Firstly, prognostic factors must be identified. These are characteristics, whether they be biomarkers, symptoms or behavioural and psychosocial factors, that among people with a given start point, are associated with, whether directly or indirectly, a subsequent endpoint [12]. These factors can not only already give clues towards modifiable targets, but can then also be combined within a prognostic model in order to predict individuals’ risk of a specific endpoint [13]. After development (and therefore internal validation), prognostic models should then be externally validated and ideally analysed for their impact in clinical practice; however, despite many models being elaborated, few are studied for their external validation and even less for their utility and influence on decision making and patient outcomes [13].

An objective and reproducible prognostic model, which includes 1 occupational, 6 biomedical, and 12 psychosocial factors, has been developed and externally temporally validated at 3 different follow-up time points, to predict RTW status: the Wallis Occupational Rehabilitation Risk (WORRK) model [14, 15]; the formula is accessible by following the link beside the reference). This model, applied at admission to rehabilitation, predicts non-return to work status following discharge from the rehabilitation centre at 3, 12 and 24 months and is applicable to a wide range of musculoskeletal injuries and patients, including those with poor health literacy or language fluency. Such a tool may aid clinicians working in physical and vocational rehabilitation centres in order to stratify patients, allowing them to be more rapidly screened and put into programmes best suited to their likely return to work outcome and therefore improving the efficiency of vocational rehabilitation. The purpose of this study was therefore to evaluate the clinical impact on decision making of the WORRK prognostic model, by analysing if the knowledge of the risk of non-return to work (estimated by the means of the WORRK model), influences the decision to allocate patients to different physical and vocational rehabilitation programs, without jeopardizing their satisfaction regarding their rehabilitation stay.

## Materials and methods

### Design

This was a single centre, parallel group, randomised controlled trial with stratified block randomisation.

Amendments to the protocol: The non-return to work follow up at 3, 12 and 24 months, as described in the protocol for the secondary outcomes, is still ongoing and has not been included in this publication. Similarly, only participant’s socio-demographic data is included in this publication, and not data concerning the other questionnaires and function tests mentioned in the protocol. With regards to patient satisfaction, because we were more interested in outcome satisfaction and not process satisfaction, it was decided to deviate from the originally proposed satisfaction scale, and instead use the Global Impression of Change Scale (at discharge compared to at admission). The CONSORT checklist (S1 File), the Project protocol (S2 File) and the data for the primary analyses (S3 File) are provided as supporting information.

### Participants

The setting of this trial was the “Clinique Romande de Réadaptation” (CRR), a Swiss rehabilitation medical centre financed by the main accident insurance in Switzerland (SUVA). Patients, mostly blue collar workers, half of whom are immigrant workers, are referred by insurance medical advisors, orthopaedic surgeons or general practitioners, predominantly between 9 to 12 months after mostly traffic and work accidents with orthopaedic trauma of the back, upper or lower limb as well as multiple traumas, if they exhibit persistent pain and functional limitations. Multidisciplinary therapeutic programs are put in place in order to improve functional status, quality of life, and the chance of returning to work. We included patients that had no severe traumatic brain injury at time of accident (Glasgow coma Scale >8), had no spinal cord injury, were capable of judgment, were not under legal custody and were not younger than 18 or older than 60 years of age at the time of rehabilitation. Most of the patients were blue collar workers and were injured after traffic, work or leisure accidents [8, 16].

### Description of the clinical pathways

Each patient admitted to the CRR is during his or her first week, allocated to one of three rehabilitation pathways. Patients can be transferred from one pathway to another over the course of the rehabilitation.

The Simple Pathway (for patients with a low risk of not returning to work) provides individual and group physiotherapy for reduction of impairments and physical conditioning (16–18 hours/week on average) of which there are 4–6 hours/week of training in vocational workshops with an average duration of 5 weeks rehabilitation. There are generally no psychosocial interventions.

The Coaching Pathway (for intermediate risk profiles) is composed of a similar schedule to the previous Pathway (in terms of type and number of hours/day of therapy and average stay), but integrates cognitive and behavioural therapies (individual and/or in groups by means of four sessions throughout the rehabilitation) and often assessment of social conditions (including insurance aspects and social advice) by social workers and occupational psychologists.

The Evaluation Pathway (for high risk profiles) comprises mainly grou6p physiotherapy sessions and vocational workshops are two hours long at most (total of 12–14 hours/week) with rehabilitation being on average 3 weeks. The main goal is to clarify the medical situation and the residual functional capacities. Psychological and social assessments are only planned if needed.

### Intervention: The WORRK model

The WORRK model was completed for all patients (control and intervention), by a team of trained nurses, giving an individual probability (expressed in %) of non-return to work. Clear instructions as to how investigators should answer the different items are available, and the predictive formula is programmed on electronic devices (reference already mentioned in the introduction). This score was then revealed, for only the intervention group, to the medical doctors before their decision as to which clinical pathway the patient should be allocated. Guidelines where provided for interpretation, including the study’s objectives and recommendations for use (probability score under 50% of nRTW, “Simple” or “Complex pathway” are probably most suitable, 50–69% of nRTW the “Evaluation Pathway” should be considered, over 70% of nRTW, the “Evaluation Pathway” is probably the most suitable choice). Several specifications were also given: the probability score was for not returning to work and therefore the higher the score, the lower the chances are of returning to work, the score was only a prediction and did not represent the exact future of the patient, and the clinical pathway choice remained the clinician’s taking into account his or her impressions and the context of the patient.

### Control

The only difference for the patients in the control group was that the corresponding medical doctor and the rehabilitation team did not receive the information from the WORRK model.

### Outcomes

The primary outcome was the proportion of patients allocated to the Evaluation Pathway. The number of patients allocated to the three different pathways was be gathered by the administrative planning unit. Furthermore, we analysed the number of transfers of pathway allocation during the course of each rehabilitation stay.

The secondary outcome was the patient’s satisfaction, measured by the Global Impression of Change Scale at discharge (compared to at admission). This scale shows patients’ beliefs concerning the importance of their improvement or worsening, and thus the efficacy of the treatment, and is recommended by the Initiative on Methods, Measurement, and Pain Assessment in Clinical Trials, for use in chronic pain clinical trials as an outcome measure [17].

### Sample size

This randomised controlled trial was designed as a parallel group, superiority trial with one primary outcome, the proportion of patients allocated by the team to the Evaluation Pathway. Today, only 10% of all patients are allocated to the Evaluation Pathway, therefore we assumed that the proportion allocated to this pathway in the control group would be 0.1. We assumed that an improvement of the allocation rate of 15% was the minimally clinically important difference (from 10 to 25% on the Evaluation Pathway). Setting the type-I error rate at 5% and the statistical power to 80% and using a two-sided Z-Test, we needed to include 112 patients per group (for details see: [18]). In order to allow for the estimated attrition rate of 25%, we included 280 patients.

### Randomisation

Once a patient was admitted to the clinic, a study nurse checked the eligibility criteria, informed the patient about the study (orally and in writing). All participants signed an informed consent form. The WORRK model was completed for all included patients.

The sequence list was generated with a stratified block-randomization technique (stratified for the risk score (five strata, with cut-offs at 0.2, 0.4. 0.6. 0.8 risk)). We performed the stratified block randomization with block length of random order from 2 to 8– unknown to the staff—with the user written ad-on programme *ralloc* within Stata 14.1.

The allocation list was kept at an external office. The included patients’ unique numbers were sent over a secure e-mail server to the external randomisation office and the allocated intervention was received in the same manner.

### Blinding

Patients were considered as blinded; the rehabilitation team did not communicate the score predicted by the WORRK tool. The assessors of the primary and secondary outcomes (the administrative planning unit and the patient his- or herself, respectively) were blinded to the group allocation. The statistical team was blinded during the data-cleaning period.

### Statistical methods

Descriptive statistics: Baseline characteristics of the patients for all known and potential prognostic variables were described overall and per intervention group with mean and standard deviation. Differences between groups in baseline values were described and interpreted based on clinical knowledge as well as with effect sizes (Cohen’s d for continuous outcomes; Phi for binary data, Cramers’ Phi for categorical outcomes). Effects sizes of 0.2 can be considered as small differences, 0.5 as moderate and 0.8 as large differences [19].

Primary outcome: The difference of the proportion of patients allocated to the evaluation pathway between the intervention and the control group was expressed with the risk ratio and the absolute risk difference (ARR), both with corresponding 95% confidence intervals, calculated with the cs command within Stata (Stata version 14.1, StataCorp, Texas). The cs command is a standard Stata command to calculate the ratio of two risks (i.e. intervention group and control group in our case) with exact confidence intervals. The number needed to treat (NNT) was calculated from the absolute risk difference (1/ARR). We did a sensitivity analysis where the changes in the rehabilitation pathways was taken into consideration. We did a sensitivity analysis taking into account the patients transferred to a different pathway during the rehabilitation.

Secondary outcomes: We calculated the risk ratio for the patients’ satisfaction, assessed with the evaluation of their impression of change over the course of the rehabilitation. We additionally made an analysis of the clinicians’ satisfaction with the WORRK tool after the end of the study. All statistical tests were two-sided.

## Results

**Fig 1:**
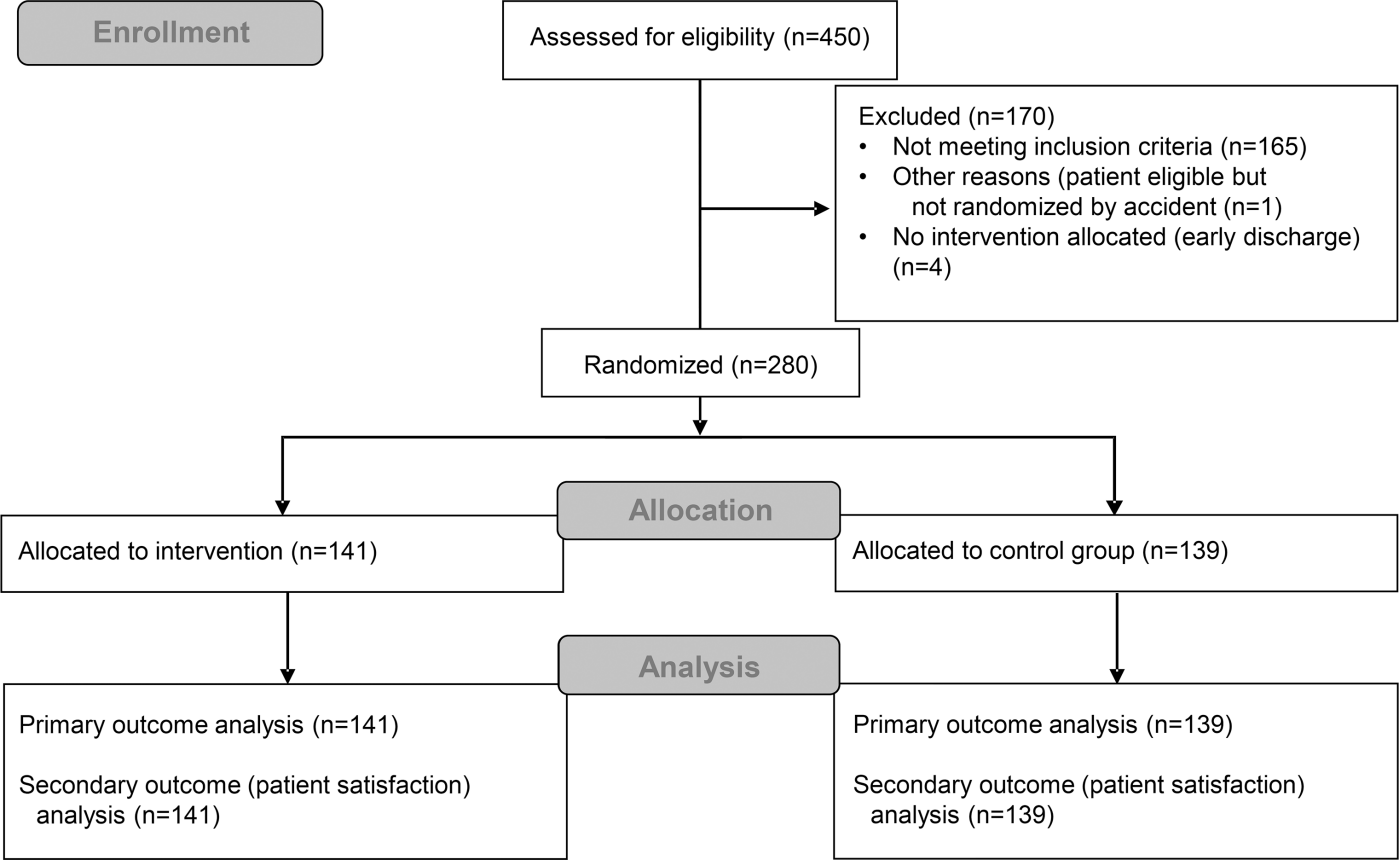
CONSORT flow diagram of the study.

We assessed 450 patients for eligibility and included 280 patients between March and November 2015, 139 being attributed to the control group (mean age 42.3 years) and 141 to the intervention group (mean age 43.2 years), with no lost data concerning the primary and secondary outcomes (see Fig 1). The two groups were very similar in regards to age, gender, pain, quality of life, probability score (estimated by the WORRK tool), education, certification, and type of accident (see Table 1).

**Table 1:** Characteristics of study participants.

	All		Intervention		Control		Between-Group
Variables	n	mean (sd) or n(%)	n	mean (sd) or n(%)	n	mean (sd) or n(%)	Effect Size
Women	280	38 (14%)	141	19 (13%)	139	19 (14%)	-0.006
Age (years)	280	42.71 (10.54)	141	43.16 (10.26)	139	42.26 (10.83)	-0.09
Pain (0 to 100)	280	50.52 (25.93)	141	49.2 (26.09)	139	51.87 (25.79)	0.10
Quality of life (0 to 100)	280	45.1 (23.27)	141	44 (23.48)	139	46.21 (23.09)	0.10
Risk not to return to work (in %)	280	60.6 (19.23)	141	60.53 (19.05)	139	60.66 (19.49)	0.01
Higher education (> 9 years)	280	115 (41%)	141	60 (43%)	139	55 (40%)	0.06
Having a professional certification	280	84 (30%)	141	43 (30%)	139	41 (29%)	0.02
Working full time	279	243 (87%)	141	125 (89%)	138	118 (86%)	0.09
Injury was declared as work injury	280	165 (59%)	141	84 (60%)	139	81 (58%)	0.03
Local language was native language	280	86 (31%)	141	41 (29%)	139	45 (32%)	-0.07

Effect size: 0.2 can be considered as a small difference, 0.5 a moderate difference and 0.8 a large difference. sd = standard deviation, n = number of participants.

### Primary outcome

**Fig 2:**
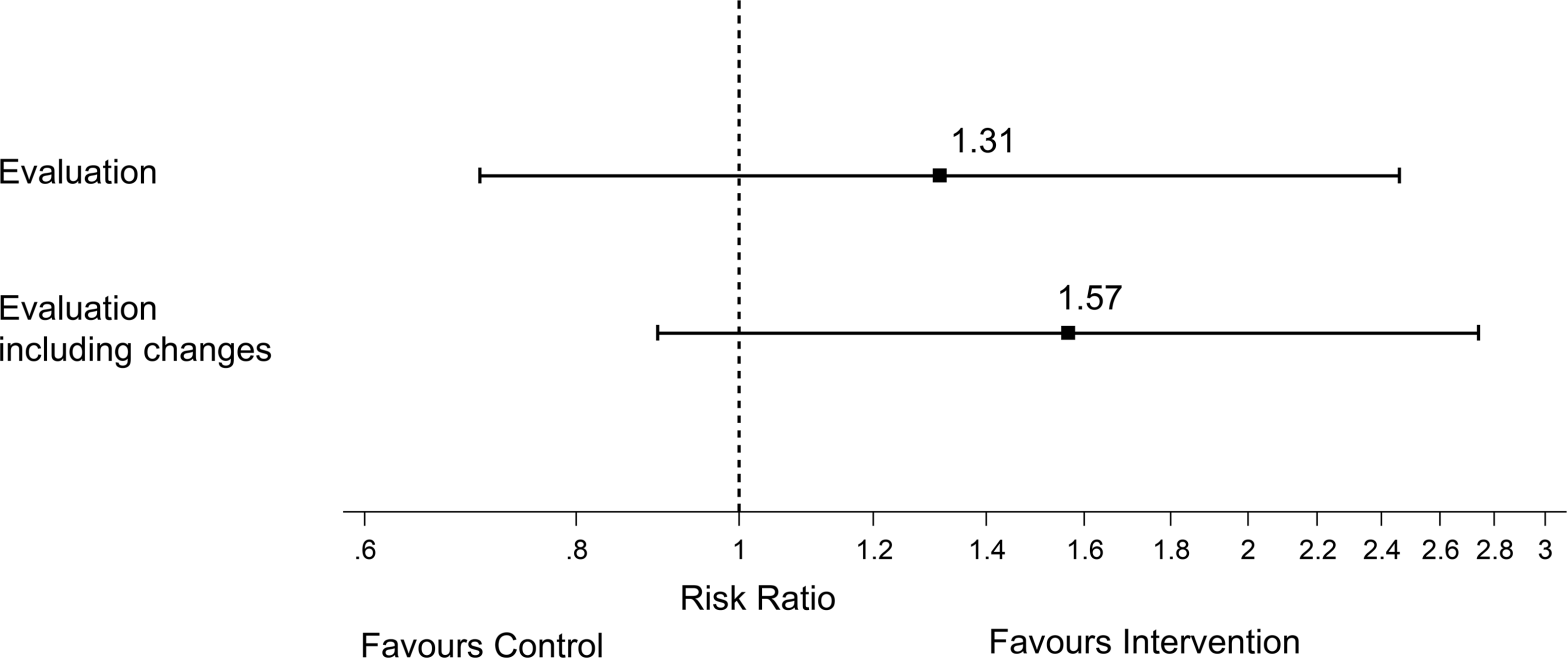
Point estimate and 95% confidence interval for the risk ratio for the referral to the “Evaluation Pathway” in the intervention group compared to the control group. The upper part shows the risk ratio for the primary analysis; the lower part shows the analysis taking into account the patients who were transferred into the “Evaluation Pathway over the course of the stay.

In the control group, 15 patients were allocated to the “Evaluation Pathway” while there were 20 allocated to this pathway in the intervention group (see Table 2). The patients in the intervention group were therefore more often referred to the “Evaluation Pathway”, having a 31% higher chance, but this difference was not statistically significant (RR 1.31 [95% CI 0.7–2.46]) (see Fig 2). The absolute risk reduction was calculated to be 3.4%, giving a NNT of 30. When taking into account the patients transferred to a different pathway during the rehabilitation, more patients from the intervention group were transferred into the “Evaluation Pathway” over the course of the rehabilitation (7 patients) than from the control group (2 patient). This increases the chances of being referred to the “Evaluation Pathway” to 57% (RR 1.57 [95% CI 0.89 to 2.74]) but again this difference was not statistically significant (see Fig 2).

**Table 2:** Allocation to the different treatment pathways.

	All			Intervention			Control		
Programme	N Allocated (%)	N End Rehab (%)	Changed (%)	N Allocated (%)	N End Rehab (%)	Changed (%)	N Allocated (%)	N End Rehab (%)	Changed (%)
Complex Pathway	208 (74.3)	199 (71.1)	-9 (-4.3)	103 (73)	96 (68.1)	-7 (-6.8)	105 (75.5)	103 (74.1)	-2 (-1.9)
Simple Pathway	37 (13.2)	37 (13.2)	0 (0)	18 (12.8)	18 (12.8)	0 (0)	19 (13.7)	19 (13.7)	0 (0)
Evaluation-Pathway	35 (12.5)	44 (15.7)	9 (25.7)	20 (14.2)	27 (19.1)	7 (35)	15 (10.8)	17 (12.2)	2 (13.3)

### Secondary outcome

**Fig 3:**
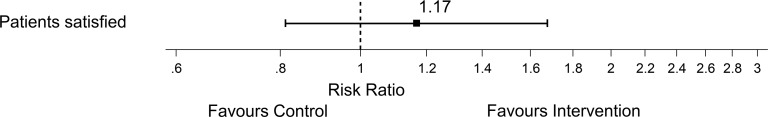
Point estimate and 95% confidence interval for the risk ratio for the patients being satisfied with the rehabilitation in the intervention group compared to the control group.

There was no decrease in the patients’ satisfaction (via the evaluation of their impression of change over the course of the rehabilitation) between the control and intervention group, with even a 17% increase in satisfied patients in the intervention group, but this difference was not statistically significant (RR 1.17 [095% CI 0.812 to 1.678]) (see Fig 3).

With regards to clinicians’ satisfaction with the WORRK tool, the qualitative analysis showed that the decision makers were mostly satisfied with the decisions they took using the WORRK score, as well as with the decision-making process it generated, and would use the tool more often if given the choice. Three of the four clinicians did not regret any decisions taken with the help of the WORRK score, and agreed that it facilitated their decision-making process. Half of the clinicians felt the WORRK score strongly influenced their decisions and that the score was came with sufficient guidelines, while the other half did not. They all strongly agreed that the WORRK score was not the only indicator they took into consideration before making their decision, and one of the four clinicians felt unsure that decisions taken using the tool were in the best interest of the patient. Additionally, it was shown that the clinicians have varying opinions concerning the “Evaluation pathway”. For example, one clinician states “there should be the possibility of having individual physiotherapy sessions for patients in the EP”, and half of the clinicians believe that by using the EP, patients are at risk of being disadvantaged while the other half do not.

## Discussion

In this randomized controlled trial evaluating the clinical impact of the WORRK model on clinicians’ decisions regarding rehabilitation program allocation, the knowledge of patient’s risk profile increased clinicians’ initial attribution to the shorter and more resource-efficient program by 31%, a result that was not, however, statistically significant or considered clinically important (according to our own power calculations). Regarding clinicians’ decision changes during hospitalization (transfer of patients to an alternate rehabilitation program), this impact is increased to 57%, still however not attaining statistical significance, with 19% of patients from the intervention group being in the “Evaluation pathway” compared with 12% in the control group. Additionally, these changes did not negatively influence patient satisfaction, with even 17% more satisfied patients in the intervention group.

The effect of the WORRK model on clinician’s behaviour is smaller than what was expected; instead of seeing a 15% increase in allocation to the “Evaluation pathway”, there was only a 3% increase when considering clinician’s initial decision and a 7% increase when taking into account decision changes during rehabilitation. Very few studies have analyzed the impact of prognostic models on clinician’s decision, and it is therefore difficult to know what effect can be judged as significant, especially considering that prognostic models vary greatly not only in their application and possible consequences on decisions, but also in the structure and guidelines accompanying them. For example, a study analyzing the impact of social interventions, which are simply suggestive, on clinician’s decisions, shows a similarly low impact (decrease in x ray prescription in chronic back pain patients (OR 1.6 [95% CI 1.1–2.3]) and decrease in rest prescription in the same population (OR 1.6 [95% CI 1.2–2.3])) [20]. However, a study analyzing a more directive intervention with specific guidelines as to the application of the information received, demonstrated a much higher impact (back pain patients referred for further physiotherapy according to their prognosis was increased by 17%) [21]. In a more acute pathology (pulmonary embolism), risk stratification showed a high impact on allocation to greater acuity units (14% increase in patients admitted to ICU) while a lower impact was found with decisions concerning invasive interventions (3% increase in patients receiving thrombolysis, 4.5% more mechanical ventilation, 3% more vasopressor use and 7% increase in inferior vena cava filter indications) [22]. This suggests that clinician’s decision making is multi-factorial, and that risk stratification can be helpful but has varying impacts depending not only on the accompanying directives, but also on the context in which the decision is taken and the potential consequences on the patient: there is a greater impact on decisions that could be regarded as being easier, taken in a calmer setting and having less consequences on patient’s immediate health such as which unit to send the patient to, while the impact is lesser when regarding urgent decisions with consequences on patient’s immediate health status.

Knowing this, it can be argued that the WORRK prognostic model was not accompanied by sufficient guidelines: indeed, the score was given with only suggestions for decisional modifications, and perhaps if these suggestions had been more directive, a more important impact may have been seen.

As our clinician satisfaction qualitative analysis showed, however, the choice of allocation of patients to the diverse rehabilitation programs is a multi-factorial one, and the WORRK model is only a small aspect that clinician’s take into account during the decision-making process. This is also supported by the findings of Stamm *et al*, [22] as discussed in the previous paragraph.

Moreover, after evaluating the clinician’s satisfaction with the WORRK model, it has been shown that clinicians are afraid to negatively influence patient’s potential outcomes by allocating them to a rehabilitation program that provides less physical and vocational interventions, as they think more therapies will increase patient satisfaction and outcome. This leads us to the conclusion that clinician education is also important for future interventions. It is known that inpatient physical and vocational rehabilitation requires a lot of resources: not only economic, but also from a patient’s perspective, with heavy physical and psychological demands coming with an intensive and inpatient program that lasts several weeks [9, 10]. This study attempted to identify high-risk patients that should be included in rehabilitation programs using fewer resources, while leaving the opportunity for revision of the decision. Although going against health care professional’s desire to improve outcomes by providing more care may seem counterintuitive, it has been shown in numerous domains that reduced interventions in high-risk populations reduce psychological and physical stress as well as health care costs, and that these decisions can be ethically and legally just [23–27]. In chronic pain following musculoskeletal injuries, investigation and management plans (especially biomedical) are often repeated despite lack of improvement, possibly submitting patients to repetitive deception, failure and reinforcing their perceived disability, causing additional physical and psychosocial strain. Additionally, in the light of ever tightening budgets, Daniels and Sabin proposed the “accountability for reasonableness” framework in order to ensure that priority setting and decisions for the distribution of healthcare resources are fair and legitimate; in order for them to seem acceptable to stakeholders (especially to those concerned by and those making the decisions), a fair process involving sustainable practices is key, with transparency, and possibility for appeals and revisions [28–30]. The procedure used in this study seems to respect these various recommendations, encouraging further investigations into the use of the WORRK model in clinical settings.

Yet another consideration to make is the role played by the cohort of patients itself and the variability of the rehabilitation programs used on the multi-factorial nature of clinician decision-making. Indeed, it is known that the type of patient analysed in this study is at risk of having psychosocial factors impeding physical recovery [7], which the clinician will give important consideration to before choosing a clinical pathway. As psychosocial interventions and therapies are integrated into the Complex Pathway, this approach will be preferred for this type of patient, and explains why the majority of patients in this centre are allocated to it, especially when considering clinicians’ fear of disadvantaging them.

In regards to patient satisfaction, the increase in satisfaction in the intervention group compared with the control group was found to be due to patients in the simple and complex programs, while patients in the evaluation program showed no change in satisfaction. We can imagine that due to the intervention of the WORRK model, clinicians were able to better identify patients who would not respond positively to the simple and complex programs, and therefore the satisfaction of patients remaining in these programs was less diluted by non-responders. The WORRK model may therefore help to better identify non-responders to programs with many hours of physical and vocational therapies.

The first strength of this study is its design, being a randomised controlled trial. Secondly, this study analyses the clinical impact of a prognostic model, which, as already mentioned, is rare, with most prognostic models being applied without impact studies to support them [13]. Thirdly, patients were not excluded depending on their health literacy or language fluency, allowing the inclusion of a diverse and representative population of orthopaedic trauma patients, reducing selection bias due to cultural criteria [31].

The principal limitation of this study is the limited generalisation, due to the specific population that was analysed with the RCT; it would be interesting to analyse the impact of the model in a different setting and health care system (for example where compensation bodies are not available). It must be noted that the WORRK model has already been externally temporally validated, and a study is currently underway for external geographical validation. An additional limitation, as already mentioned, is that the WORRK tool was not accompanied by sufficiently directive guidelines. Further limitations include factors that could jeopardise the internal validity, including events and changes that could have occurred between the start and end of a patients’ hospital stay or of the study itself (leakage, history, maturation). These include, for example, the WORRK score becoming known concerning a patient that was originally in the control group leading to a program transfer, the evolution of a patients’ health status during hospitalisation leading to transfer to an alternate rehabilitation program regardless of the WORRK score, or a medical doctor increasing his or her experience and therefore changing his or her behaviour throughout the study regardless of the WORRK score.

Future perspectives could see the WORRK model modified in its presentation, comprising more directive guidelines for facilitated use and application. Moreover, clinician education is necessary to counter beliefs that shorter and more resource-efficient rehabilitation programs potentially disadvantage patients. In order to give weight to these arguments we put forward, a study could be carried out to prove that “less is more”, analysing functional and psychological outcomes in high risk patients (estimated by the WORRK model), half of which are in a longer and more resourceful program, the other half being in a shorter and more resource-efficient program.

## Conclusion

In conclusion, the knowledge of the risk of non-return to work, estimated by the means of a prognostic model (WORRK), has an influence but that is not statistically significant and does not attain what was previously defined by our own power calculations as clinically important (15%), on clinical decision making with regards to the allocation of patients to different physical and vocational rehabilitation programs after orthopaedic trauma, without jeopardizing their satisfaction regarding their rehabilitation stay, and in a more important manner when taking into account decision changes during rehabilitation. These findings support the multi-factorial aspect of clinician decision-making.

## Other information

The protocol was registered at ClinicalTrials.gov (NCT02396173) and this study was approved by the local ethical committee (Commission cantonale valaisanne d’éthique medical–CCVEM 047/14).

## Supporting information

S1 File CONSORT checklist. (DOC)

S2 File Project protocol. (PDF)

S3 File Data file. (CSV)
