# Supplementary material for: Impact of a non-return-to-work prognostic model (WORRK) on allocation to rehabilitation clinical pathways: A single centre parallel group randomised trial
Source: PLoS One. 2018 Aug 2;13(8):e0201687. doi: 10.1371/journal.pone.0201687 (PMC6072039; doi:10.1371/journal.pone.0201687)
Supplement: S2 File — (PDF) [file pone.0201687.s002.pdf]

The WORRK Study 1

Does the knowledge of a "non-return to work" predictive score influence vocational rehabilitation after orthopaedic trauma?

Protocol for a randomised controlled trial.

Dr. François Luthi, MER, Service de Réadaptation de l'Appareil Locomoteur, Clinique Romande de Réadaptation suvacare, Avenue Grand Champsec 90, CH-1950 Sion

Tel : +41 27 603 2175

Email : francois.luthi@crr-suva.ch

Projet de Recherche original présenté pour le « Certificate of Advanced Studies » (CAS) en Recherche Clinique, UNIL, CepiC, IUMSP

(Des aides ponctuelles ont été reçues de la part de M. Roger Hilfiker, Msc, pour les paragraphes matériel et méthodes, statistiques et data monitoring. Certaines phrases techniques sont inspirées d'un précédent protocole. M. Roger Hilfiker est aussi le principal co-auteur de l'étude publiée sur le modèle WORRK).

Summary of the research plan

Background: Orthopaedic trauma are a leading cause of long-lasting sick-leave and persistent disability**.** People suffering from persistent sick-leave often need vocational rehabilitation. Vocational programs are planned to improve the likelihood of returning to work. Physical conditioning, professional evaluation and training, as well as psychological and social interventions are the core of treatments. Efficiency of these programs is moderate and there is a scope for improvements. For instance, rehabilitation programs tailored to the individual needs and potentials are called for. But, the allocation remains difficult. Decision-supportive tools may be convenient to achieve this goal. Recently, the WORRK model was proposed to early assess the risk none returning to work for those patients. The main goal of this research is to measure if the WORRK model improve patients' allocation to different vocational programs according to their "non-return to work" risk.

Methods: vocational inpatients after orthopaedic trauma (n=280), aged between 18-60 years. Three different vocational pathways can be selected: Simple (for patients with low "non-return to work" risk, 5 weeks stay), Coaching (intermediate risk, 5 weeks), Evaluation (high risk, 3 weeks). Design: randomized-controlled trial. In the intervention group, the probability not to return to work estimated with the WORRK model will be offered to the clinician team before the allocation decisions. In the control group, the probability will also be estimated but not known by the clinician team. The primary outcome is the proportion of patients allocated to the Evaluation Pathway. Secondary outcomes: the non-return to work rate is not higher in the intervention group than in the control group (follow-up at 3, 12, and 24 months); the patients' satisfaction is not worse in the intervention group; the decisions makers' satisfaction with the information available for the decision process is better in the intervention group; the proportion of patients changing the treatment pathway during the vocational stay is not higher in the intervention group; and the calibration of the WORRK model remains satisfactory.

Relevance and impact: We expect that the WORRK model will improve the efficiency of vocational rehabilitation after orthopaedic trauma. This will due to better allocation to the vocational pathways according to the patients' risk profile. If that is the case, an increase of the shorter vocational program is expected without jeopardizing the chance returning to work and the patient satisfaction with quality of care. The ability of the WORRK model to detect patients with similar risk profiles could also strengthen the interest for this decision-supportive tool in clinical practice and trials.

Key-words: work, disability, vocational, occupational, rehabilitation, prediction, orthopaedic trauma

1. Introduction/Background

The ability to work is an essential component of the quality of life. Works ensures adequate resources for covering the essential needs of people and full participation in the society. Work ability plays also a crucial role for social status, self-confidence and personal identity[1,2]. Consequently, work disability defined as a work cessation due to illness, injuries or any other medical cause[3] may have an important impact on individuals, not only on the economic and social terms, but also in terms of people's health. For instance, being off work may be associated to anxious and depressive symptoms, as well as an increase of suicide rate[4,5]. Moreover work disability incurs also huge costs to the public health system. For instance, there are more than 40 million disabled people on working age in the European Union[3]. In the UK, the global economic burden of disability is estimated to be in excess of €115 billion, more than the UK National Health Service budget[3]. And in the Netherlands the work disability reach yearly almost 5% of the gross national product[6]. Musculoskeletal and psychiatric disorders are the main causes of inability to work in western countries[3]. Among musculoskeletal disorders, non-fatal orthopaedic trauma is a leading cause of long-lasting sick-leave and persistent disability[7,8]. Nevertheless the costs due to work disability is highly skewed. For the main Swiss insurance company (called SUVA), only a small but important fraction of clients after trauma requires specialized and multidisciplinary management in order to return to work. The latter clients represent 5% of the claims but expend about 80 % of the financial resources[9]. Interestingly, this distribution of the costs is comparable to other musculoskeletal disorders like low back pain patients[10].

Consequently helping people returning to work is a focal point for public health[3,11]

Vocational rehabilitation is the process devoted to help patients disadvantaged by illness or trauma to access, maintain, or re-establish working capacity to suitable work, but also to identify those requiring disability pensions[3]. Vocational rehabilitation includes medical care as well as non-medical interventions through insurance, employment or social services[12]. The medical part of vocational rehabilitation consists of multidisciplinary functional restoration programs which includes physical, psychological, educational, social, and occupational interventions. The efficiency of medical vocational rehabilitation is generally accepted[3,13-17] for patients with chronic pain disorders, even though differences in managements and health care systems make it difficult to interpret findings[1].

In Switzerland for instance, people suffering after accidents with persistent sick-leave are often hospitalized in the two clinics that belong to the SUVA, one located in the French-speaking part of the country (Clinique Romande de Réadapation), and the second in the German-speaking part (RehaKlinik Bellikon). In total about 3000 patients per year are hospitalized for rehabilitation

programmes in both clinics. Among those, vocational rehabilitation after orthopaedic trauma represents the main objective of the patient's management[18]. Most of these patients are blue collar workers and were injured on work, traffic or leisure accidents[19]. The aim of the stay is to control the diagnosis, to improve the likelihood of returning to work (usual or adapted to impairments) by the mean of an interdisciplinary bio psychosocial approach including vocational assessment, physical conditioning and care to reduce pain and disabilities[20]. These multidisciplinary functional restoration programs take several weeks (5 weeks on average) and are costly (about 5'000 CHF per week). The success rate (i.e. return to the same or accommodated job) of this approach is 50% in both Swiss Clinics[19]. In other words 50% of recipients of vocational rehabilitation after trauma are unable to return to work despite this approach. One possible explanation for this moderate successful rate may probably be the prevalence of non-modifiable prognostic factors among recipients of VR (age, absence of certified education, non-native speaker among vulnerable workers, and cancellation of contract of employment …). Nevertheless, there is still scope to improve vocational rehabilitation. For instance, better knowledge of patients' risk profile may be a vector for improved programs, tailored to the individual needs and rehabilitation potentials.

Vocational rehabilitation seems to be an effective intervention for helping people with chronic musculoskeletal complaints (e.g. low back pain patients, trauma patients) returning to work. Nevertheless, there is a potential to increase efficiency in vocational rehabilitation.

Injured people who need vocational rehabilitation greatly vary and factors influencing return to work may also be different [21,22]. After orthopaedic trauma, known prognostic factors are somatic (like age, injury severity, pain, physical capacity...), psychological (depressive symptoms, pain attitudes, beliefs in recovery...) and social (education, social vulnerability...) [11,23-27]. Moreover, the cultural background and individual representations associated to rehabilitation may also be different among patients. For instance, non-native workers, a growing segment of the work force, are a vulnerable population at risk for poor compliance in vocational programs [28] and also being exposed to adverse working conditions [29,30]. In many researches, immigrant workers which are not fluent in native language are simply excluded, which constitutes a severe bias [31]. Having evaluation tools not relying on language fluency is important [20,32]. Moreover, it could also be interesting to customize vocational rehabilitation programs as soon as possible to the patient's needs and potentials based on the individual risk profile [33]. Finally, for those with poor prognosis of return to work, avoiding ineffective, inappropriate, and costly vocational programs is also a key-point.

In the Clinique Romande de Réadaptation, 3 different clinical pathways were established 2 years ago in order to address these concerns: 1. a Simple Pathway (5 weeks on average) for patients who need only usual vocational programs because they have only weak or no adverse bio psychosocial prognostic factors. 2. a Coaching Pathway (5 weeks on average) for people with moderate adverse prognostic factors who need intensive multidisciplinary (i.e. also psychological and social interventions) vocational program. 3. an Evaluation Pathway (3 weeks on average) for people with strong adverse prognostic factors who are unlikely to RTW even with an intensive vocational rehabilitation program. Patients are allocated to one of the pathways by the interdisciplinary team (supervised by a clinical occupational physician) after evaluation of the patients' bio psychosocial complexity [20,34,35], pain assessment [36]) and a carrying load test from floor to waist (the PILE test [37]). To date, only 10 % of the patients are allocated to the Evaluation Pathway, although about 50% of all patients are unsuccessful to RTW after vocational programs. Conversely, 60% of patients are allocated to the Coaching Pathway (5 weeks) that consumes the most therapeutic resources. This unsatisfactory result has also a cost for the insurance system: the length of stay is too long for many people. Therefore, the number of patients who could have benefitted from vocational rehabilitation is diminished (for instance, hundreds of people each year are waiting before entering into vocational programs in the Swiss Trauma clinics).

Factors influencing the allocation decision are unclear. The lack of appropriate decision-supportive tool available during the allocations' treatment period could be one of the reasons for this. In cases of doubt, clinicians possibly prefer a more conservative approach to give patients the best chance of being successful (i.e. ability returning to work). Moreover, the inability to precisely and rapidly allocate patients with similar risk of non-RTW may also hamper the development and testing of new and alternative vocational approaches for helping vulnerable people [38-40].

In other words, people in charge of vocational rehabilitation need appropriate decision-supportive tools to early estimate the likelihood of non-return to work, suitable for all kinds of trauma and among all patients, irrespective of their social and cultural backgrounds. Appropriate decision-supportive tools may help to improve efficiency of vocational rehabilitation as well as the design of clinical trials.

So far, prognostic models assessing return to work after orthopaedic trauma were only designed for the acute phase [41-43] and consequently were not suitable for the vocational phase. Recently, based on a prospective cohort of over 2000 patients, we developed and validated a simple (19 items) prognostic model to assess the risk of non-returning to work for those patients [32]: the WORRK

model. This WORRK model is an observer rated tool. It has acceptable discrimination performance (AUC 0.73, 95% CI 0.70 to 0.77) and good calibration. It is suitable to all kinds of trauma and also to vulnerable population with poor language fluency and/or health literacy, like immigrant workers [31,32]. Fill the form takes less than 20 minutes and a predictive score may be electronically calculated. But, to date the clinical usefulness of this model was never tested.

The main hypothesis driving this research is that early knowledge of the "non-return to work" predictive score, by the mean of the WORRK model, will influence the allocation to the 3 different Clinical Pathways. In other words, we plan to test if a greater proportion of people entering in vocational rehabilitation will be allocated to the Evaluation Pathway if the risk of non RTW by the mean of the WORRK model is known by the clinicians in charge. And we also plan to test if this new approach is not harmful for the patients, i.e. if it doesn't compromise the chance to return to work.

Main Research Question (Primary Outcome)

Does the knowledge of the risk of non-return to work, estimated by the means of the WORRK model, influence the decision to allocate patients to the Evaluation Pathways program after orthopaedic trauma, without jeopardizing the chance of returning to work?

Secondary Research Outcomes

1. The non-return to work rate is not higher in the intervention group than in the control group in a margin of less than 3 % [14] (non-inferiority trial).
2. Patient's satisfaction is not worse in the intervention group than in the control group in a margin of less than 10 % (non-inferiority trial)
3. The decisions makers' satisfaction with the information available for the decision process is better in the intervention group.
4. The proportion of patients changing the treatment pathway during the vocational stay is not higher in the intervention group in a margin of 5% (non-inferiority trial).
5. The calibration and discrimination of the WORRK model remains satisfactory (intercept, the Hosmer-Lemeshow test, and the AUC ≥ 0.7).

2. Material and Methods

2.1 Study design:

Randomized controlled trial

2.2 Setting and participants:

This study will take place in the Clinique Romande de Réadaptation (CRR) at Sion (Canton of Wallis), one of the two Trauma Swiss clinics, which is located in the French-speaking part of Switzerland. All patients hospitalized for a vocational rehabilitation program after orthopaedic trauma will be eligible for this study (more than 600 patients each year). Patients are mostly blue collar workers, with orthopaedic trauma of the back, upper or lower limb and multiple traumas [19]. The median time from accident to rehabilitation is 9 months. Patients are referred to the clinic from all of the French speaking cantons of Switzerland, which includes urban and industrial city centres like Geneva or mountainous and more rural regions like Wallis. Switzerland is also a country with an important proportion of immigrant workers in all sectors of the economy (see for details www.bfs.admin.ch/bfs/portal/fr/index) and 50% of patients addressed to our vocational facility are immigrant workers [19,20,32].

2.3 Process description of the clinical pathways:

1. The Simple Pathway (for low risk of non-returning to work) provides individual and group physiotherapy for reduction of impairments and physical conditioning (2.5 hours/day on average), training in vocational workshops (from to 2 to 4 hours/day). No specialized psychological, like cognitive therapies, are planned. Social evaluations are only planned according to specific needs. The treatment time is planned to progressively correspond to at least a half-time work volume (i.e. 4 hours a day). The duration of this pathway is on average 5 weeks. At the end of the hospitalization a return to work schedule is proposed (same or accommodated job), and, if needed complementary therapeutic or occupational modalities are provided.
2. In The Coaching Pathway (for intermediate risk of non-returning to work), the main difference is that psychological cognitive therapies are integrated in the care (individual and/or in group). Assessment of social conditions (including insurance aspects) by social workers and occupational psychologist are also made in most cases. Treatment time (including vocational workshops) and the length of stay are similar to the Simple Pathway (on average 5 weeks). At the end of the hospitalization a return to work schedule is also proposed (same or accommodated job), and, if needed complementary therapeutic or occupational modalities are also provided.
3. In the Evaluation Pathway (for high risk of non-returning to work), physiotherapy is mainly given in a group format. Vocational workshops are two hours long at most. The main goal is to clarify the medical situation and the residual functional capacities. Psychological and social assessments are only planned if needed. There is generally no further role for

complementary therapeutic or occupational modalities at the end of the hospitalization. A remaining ability to work is estimated. The duration of this pathway is on average 3 weeks.

2.4 Intervention: the WORRK model

The WORRK model [32] is a predictive tool (19 items) of the non-return to work risk useful for all kinds of orthopaedic trauma and for patients needing vocational rehabilitation. It is constructed with variables independent of the patient's education and language fluency. It is a short patient's bedside tool and takes less than 20 minutes. Clear instructions on how investigators should answer to the different items are available, and the predictive formula is programmed on electronic devices. For details, see the supporting information (supplementary). The WORRK tool will be filled in for all patients, even the patients in the control group. However, the prediction tool result will only be accessible for the medical doctors and the team of the patients in the intervention group, with the guidelines for the interpretation ("1. With a probability score over 50% (not to return to work), patient's allocation to the "Evaluation Pathway" should be considered" "2. With a probability score over 70% (not to return to work), the "Evaluation Pathway" is probably the most suitable choice").

2.5 Data

2.5.1 Socio-demographic data

The baseline socio-demographic data will be collected including the participant's gender, age, marital status, educational attainment, current work status, time since their injury, as well as the injury severity score measured according to the Abbreviated Injury Scale (AIS)[44], (rank 1 to 5; 6 = fatal injury), and the localization of the lesion. At the end of the hospitalization the patients will answer to the Global Impression of Change Scale to assess change over time Comparing to entry, the patients will be asked if their pain associated disabilities had changed on a 7 point scale (1 = worse than ever; 2 = much worsened; 3; slightly worsened, 4 = unchanged, 5 = slightly improved; 6 = much improved; 7 = completely improved). The score will be analysed in 3 groups: worsened (1 or 2), unchanged (3-5) or improved (6-7)[45].

2.5.2 Questionnaires and function tests

The questionnaires used in our daily practice will also be collected (Brief Pain Inventory [36], Tampa Scale for Kinesiophobia [46], Pain Catastrophizing scale [47], Hospital Anxiety and Depression scale [48], Spinal Function Sort [49] (for spinal and lower limb injuries) or the Hand Function Sort [50] (for upper limb injuries), and also questions on patient's expectations about recovery, perceived lesion severity, and quality of life (VAS). The results of the usual function tests will also be collected: 6'

walking test, five sit-to-stand test, 1’ stairs climbing test, PILE test, grip strength with the JAMAR dynamometer [37,51,52].

2.6 Patients:

We will randomize 280 consecutive patients attending the Clinique romande de réadaptation (CRR SUVA, Sion, Switzerland).

2.6.1 Sample Size calculation:

This randomised controlled trial is designed as a parallel group, superiority trial with one primary outcome, the proportion of patients allocated by the team to the Evaluative Pathway. Today, only 10% of all patients are allocated to the Evaluative Pathway, therefore we assume that the proportion allocated to the Evaluation Pathway in the control group will be 0.1. We assume that an improvement of the allocation rate of 15% is the minimally clinically important difference (from 10 to 25% on the Evaluation Pathway). Setting the type-I error rate at 5% and the statistical power to 80% and using a two-sided Z-Test, we would need to include 112 patients per group (for details see: Designing Clinical Research, 4th edition, Hulley et al. page 75, Lippincott 2013 [53]). In order to allow for the estimated attrition rate of 25%, we will include 280 patients.

2.6.2 Inclusion criteria

- Patients hospitalized for a vocational rehabilitation programme after an orthopaedic trauma
- Aged 18 to 60 years

2.6.3 Exclusion criteria

- Severe traumatic brain injury at time of accident (Glasgow coma Scale ≤8).
- Spinal Cord Injury
- Not capable of judgment
- Under legal custody

2.6.4 Allocation:

Once a patient is admitted to the clinic, a study nurse will check the eligibility criteria, inform the patient about the study (orally and in written). If the patient signs the informed consent form, the study nurse will call a central randomization center (not at the clinic) who will provide the group allocation after registration of the patient. The WORRK tool will be filled in for all patients, even the

patients in the control group. However, the prediction tool result will only be accessible for the medical doctors and the team of the patients in the intervention group.

(a) **Intervention Group (Group A):** The rehabilitation team receives the results from the WORRK tool with the guidelines for the interpretation (see above, p. 8).

(b) **Control Group (Group B)**: The rehabilitation team does not receive the results from the WORRK tool. The patient's allocation is made as usual.

2.6.5 Generation of random allocation sequence:

The sequence list will be generated with a stratified block-randomization technique (stratified for the risk score (five strata, with cut-offs at 0.2, 0.4. 0.6. 0.8 risk). We will perform the stratified block randomization with block length of random order from 2 to 8– unknown to the staff - with the user written ad-on programme *ralloc* within STATA 13.1 (or newer version if available).

2.6.6 Allocation concealment:

The allocation list will be kept at an external office. The study nurse of the clinic will, after receiving the signed informed consent, either phone to the external office or write an email over a secure e-mail server to the external office with the name of the participant and will receive the allocated intervention back by e-mail or on the phone.

2.6.7 Blinding:

Patients will be considered as blinded; the rehabilitation team will not communicate the result of the WORRK tool. The blinding of the rehabilitation team will not be feasible. The assessor of the primary outcome and the fourth secondary outcome (the administrative planning unit) is blinded to the group allocation. The assessor of the first two and the last secondary outcomes (in this case the patients) are also blinded. The assessor of the third secondary outcome (the decisions makers) cannot be blinded. The study nurse who sends the questionnaire and contacts the patient in case of questions or non-return of the questionnaire will be blinded. The statistical team will be blinded during the data-cleaning period.

2.7 Overview of the protocol

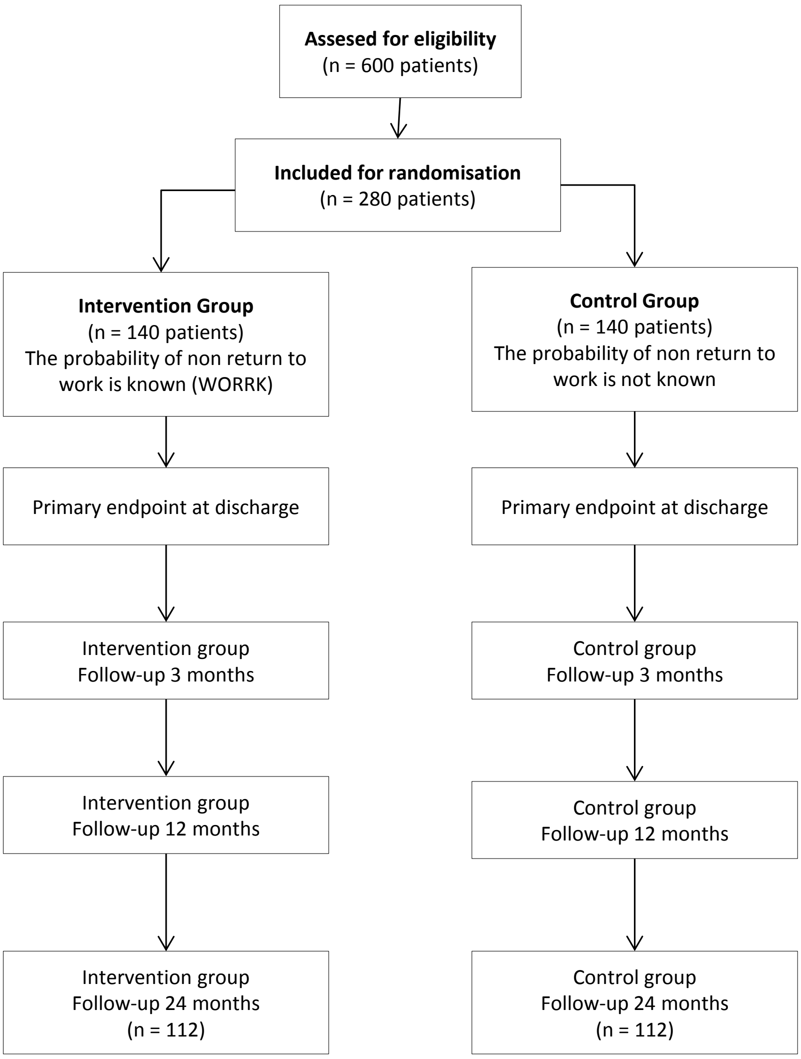

Figure 1: planned study flow chart

2.8 Training of study nurses and rehabilitation team:

Before the start of the study, all involved persons who will have to inform or assess any kind of patient data related to the study are trained in four two-hour workshops.

2.9 Outcome measures

2.9.1 Primary Outcome:

The primary outcome in the WORRK study is the proportion of patients allocated to the Evaluative Pathway. The number of patients allocated to the 3 different pathways (intervention and control group) will be gathered by the administrative planning unit. Furthermore we will analyse if there is a shift of the allocation pattern over time.

2.9.2 Secondary Outcomes:

All the questionnaires are already available in most of the foreign languages spoken in the clinic (Portuguese, Italian, Spanish, Albanian, Serbian…)

1. The non-return to work rate will be assessed by questionnaire at 3 months, 12 months and 24 months after discharge. Patients will be asked about work status and the date of work resumption if applicable. Patients working at full or part time, same or accommodated job (at least half time), will be considered as working. In case of non-response, two reminders will be sent. If necessary patients will be contacted by telephone or email.
2. Patient's satisfaction will be measured at discharge by five items using a 5-point Likert scale ("poor", "fair", "good", "very good", and "excellent"). These questions[54] explore: "Explanations about what will be done to you during treatment"; "Quality of information you received at the end of the treatment regarding future"; "Feeling of security at all times during treatment"; "Extent to which treatment was adapted to your problem"; "Would you recommend this facility to people close to you".
3. The decisions makers' satisfaction with the information available for the decision process will be measured by a Visual Analogue Scale from 0 to 100 (from "not satisfied at all" to "100% satisfied"). Furthermore we will analyse if there is a shift in the satisfaction over time in the intervention group (learning curve of the ease of use of the probability score between start and end of the trial).
4. "Cross-over patients" (i.e. patients transferred in another pathway during rehabilitation) will be gathered by the administrative planning unit.
5. The calibration of the WORRK model will be checked by remains satisfactory (intercept, p-value of The Hosmer-Lemeshow, and the AUC ≥ 0.7).

3 Data management

3.1 Missing Data

In order to reduce missing values, we will encourage patients who want to stop the interventions to allow us to assess at least the return to work status at three months, one year and two year follow-up. To reduce the missing data, we will ask the patients': a. postal address b. phone number c. mobile phone d. email address. To allow for a true intention to treat analysis, the study coordinator will be in charge to reduce missing values and where necessary implement and adapt strategies for data acquisition and documentation. Data will be entered in directly on electronic forms with a digital pen system or scanned from paper-versions (follow-up questionnaire). If the follow-up

questionnaire is not returned, one reminder will be sent by post or e-mail. If there is still no return, a blinded assessor will contact the patients per phone to receive the information.

Data will be exported to STATA in anonymised form.

For the sensitivity analysis, we will perform multiple imputations of the missing values using an iterative process with prediction models for each variable with missing values with chained equations in STATA.

3.2 Statistical Analysis

Descriptive statistics: Baseline characteristics of the patients (all known and potential prognostic variables will be described overall and per intervention group with mean, standard deviation and minimal and maximal values if appropriate or with median, 25th and 75th percentiles as well as minimal and maximal values. Differences between groups in baseline values will be described and interpreted based on clinical knowledge as well as with effect sizes (Cohen's d for continuous outcomes; Phi for binary data, Cramers' Phi for categorical outcomes).

3.2.1 Analyses of primary outcome:

The data for the primary outcome at the primary endpoint (allocation rate of the two arms) will be analysed with a Z-Test with p-value and a corresponding 95% confidence interval. In a secondary analyses, we will perform a logistic regression with the dependent variable "allocated to the Evaluation Pathway" and intervention or control group as independent variable. First we will present a crude odds ratio for the odds of being allocated to the Evaluation Pathway when the rehabilitation team knows the WORRK (intervention group) versus the rehabilitation team not knowing the WORRK risk score (control group).

3.2.2 Analyses of secondary outcomes:

1. *Return to work rate*

To test the hypothesis that the non-return to work rate in the intervention group is not higher than in the control group (non-inferiority trial) we will use a one-sided test with a significance level of alpha 0.025 and a non-inferiority margin of 3% [14].

2. *Patient's satisfaction*

To test the hypothesis that the patients' satisfaction is not worse in the intervention group than in the control group (non-inferiority) is evaluated with a one-sided test with a significance level set at

0.025 and a non-inferiority margin of 10%

3. *Decision makers' satisfaction*

To test the hypothesis that the decision maker's satisfaction is better in the intervention group than in the control group (superiority) is evaluated with a two-sided test with a significance level set at 0.05.

4. *Crossing treatment groups*

To test the hypothesis that in the intervention group not more patients are changing the treatment (e.g. the rehabilitation team decided after two weeks that a patient who received the Evaluative pathway should receive the Coaching pathway) than in the control group (non-inferiority) is tested with a one-sided test with a significance level set at 0.025 and an non-inferiority margin of 5%.

5. *Calibration of the WORRK model*

The calibration at large will be assessed with the intercept of the prediction model (H0 intercept = 0) and the calibration will be assessed with a calibration plot (H0 slope = 1) with corresponding 95% confidence intervals and a Hosmer-Lemeshow test.

Discrimination will be assessed with the area under the receiver operating characteristic curve.

3.3 Intention to treat

All analysis will be performed as (a) true intention to treat analyses with imputation (multiple imputation methods) of missing values, and (b) as "available data intention to treat analysis" with intention to treat analysis of all available (i.e. non-missing) values. Multiple imputations will be performed within STATA version 13.1 or newer version.

3.4 Data Monitoring

Decision to terminate the trial:

Intermediate analysis will be performed by an independent statistician but not reported to study members and decision on the termination of the study will not be based on intermediate results (e.g. between group or pre-post effect sizes). The intermediate analyses will be used as a quality control. The study would only be terminated early if (a) significant accumulation of adverse event in the experimental group occurs (statistical significance will be based here on a cumulative Bayesian method), or (b) if inclusion of patients is not possible within the given time frame.

Supervision of data collection process:

A scientific collaborator will supervise the data-collection process and control intermediate data regularly (at least once a month). Only persons blinded to intervention allocation will have access to the trial data during the trial (e.g. blinded statistician, study nurse or ethical committee). The blinding will be lifted only once data cleaning was performed.

4 Ethical Issues:

The project will be submitted to the cantonal ethical committee (canton Valais, Switzerland). All data will be anonymised. The key to link trial data to actual patients name is stored in a database to which only persons with specific rights have access.

Before being admitted to the clinical trial, patients must consent to participate after the nature of the clinical trial and possible consequences have been explained. Patients must give written informed consent to participate in the study.

We expect a very low risk for adverse events. Treatments will be made as usual according to the existing Pathways. Nevertheless, all the adverse events reported by participants or members of the clinical team will be collected during the trial and will be documented. As mentioned above, the study would be terminated early if significant accumulation of adverse event in the experimental group occurs.

5 Publication policies:

Results will be published as stated in the protocol, which will be registered before the start of the trial. No third party can influence the decision on publication. The applicant has the full responsibility on the publication of the data.

6 Timeline of the study

We will need 3 months to set-up the study (ethical commission, preparation of information material, data base…). As we treat 600 eligible patients per year and we assume that 75% patients accept to participate, we hope to include between 35 and 40 patients per months. Therefore, about 8 months will be necessary to include all 280 patients. The follow-up time is planned during 24 months.

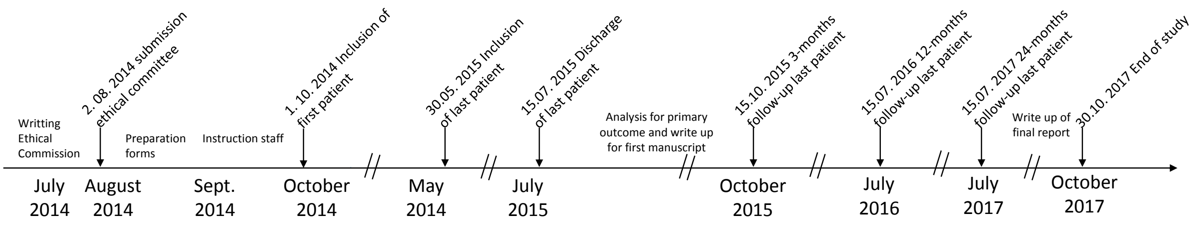

Figure 1: Timeline with milestones

7 Budgets

**Table 1:** Financial charges in addition to usual care, due to the study		
**Human resources**		
Research assistant	August 2014 – July 2015 (20%), 1y	20’000 CHF
	August 2015 – July 2017 (10%), 2y	20’000 CHF
Epidemiologist	80 hours	10’000 CHF
**Other financial charges**		
Ethical committee		1’500 CHF
Mailing (postal)		3’000 CHF
Congress		2’000 CHF
Publication fees (op access J)		3’000 CHF
**Total amount**		**59’500 CHF**

8 Relevance and impact

Predictive models and other decision-supportive tools have only the potentials to influence clinical practice if their usefulness may be proven [55,56]. We expect that the WORRK model [32] will improve the efficiency of vocational rehabilitation after orthopaedic trauma due to better allocation to the different vocational pathways. If that is the case, an increase of the shorter vocational programs is expected without jeopardizing the chance returning to work and the patient satisfaction with quality of care. The number of unnecessary long and costly vocational stays will also be reduced what also presents an opportunity to reduce the vocational waiting lists. The ability of the WORRK model to detect patients with similar risk profiles could also strengthen the interest for this decision-supportive tool in clinical practice and trials.

Appendix: supportive information for the WORRK model**;** patient’s information and informed consent form.

8 Bibliography

1. Gobelet C, Franchignoni F (2006) Vocational Rehabilitation. Paris: Springer-Verlag. 403 p.
2. Stigmar KG, Petersson IF, Joud A, Grahn BE (2013) Promoting work ability in a structured national rehabilitation program in patients with musculoskeletal disorders: outcomes and predictors in a prospective cohort study. BMC Musculoskelet Disord 14: 57.
3. Chamberlain MA, Fialka Moser V, Schuldt Ekholm K, O'Connor RJ, Herceg M, et al. (2009) Vocational rehabilitation: an educational review. J Rehabil Med 41: 856-869.
4. Milner A, Page A, LaMontagne AD (2013) Long-term unemployment and suicide: a systematic review and meta-analysis. PLoS One 8: e51333.
5. Rosenthal L, Carroll-Scott A, Earnshaw VA, Santilli A, Ickovics JR (2012) The importance of full-time work for urban adults' mental and physical health. Soc Sci Med 75: 1692-1696.
6. Noben CY, Nijhuis FJ, de Rijk AE, Evers SM (2012) Design of a trial-based economic evaluation on the cost-effectiveness of employability interventions among work disabled employees or employees at risk of work disability: the CASE-study. BMC Public Health 12: 43.
7. Lyons RA, Kendrick D, Towner EM, Christie N, Macey S, et al. (2011) Measuring the population burden of injuries--implications for global and national estimates: a multi-centre prospective UK longitudinal study. PLoS Med 8: e1001140.
8. Black JA, Herbison GP, Lyons RA, Polinder S, Derrett S (2011) Recovery after injury: an individual patient data meta-analysis of general health status using the EQ-5D. J Trauma 71: 1003-1010.
9. Morger W (2008) Vocational rehabilitation in the Swiss social insurance system - SUVA's solution. J Rehabil Med 40: 6-7.
10. Wieser S, Horisberger B, Schmidhauser S, Eisenring C, Brugger U, et al. (2011) Cost of low back pain in Switzerland in 2005. Eur J Health Econ 12: 455-467.
11. Kendrick D, Vinogradova Y, Coupland C, Christie N, Lyons RA, et al. (2012) Getting back to work after injury: the UK Burden of Injury multicentre longitudinal study. BMC Public Health 12: 584.
12. Gobelet C, Luthi F, Al-Khodairy AT, Chamberlain MA (2007) Vocational rehabilitation: a multidisciplinary intervention. Disabil Rehabil 29: 1405-1410.
13. Lambeek LC, Bosmans JE, Van Royen BJ, Van Tulder MW, Van Mechelen W, et al. (2010) Effect of integrated care for sick listed patients with chronic low back pain: economic evaluation alongside a randomised controlled trial. BMJ 341: c6414.
14. Squires H, Rick J, Carroll C, Hillage J (2012) Cost-effectiveness of interventions to return employees to work following long-term sickness absence due to musculoskeletal disorders. J Public Health (Oxf) 34: 115-124.
15. Hoefsmit N, Houkes I, Nijhuis FJ (2012) Intervention characteristics that facilitate return to work after sickness absence: a systematic literature review. J Occup Rehabil 22: 462-477.
16. Schaafsma FG, Whelan K, van der Beek AJ, van der Es-Lambeek LC, Ojajarvi A, et al. (2013) Physical conditioning as part of a return to work strategy to reduce sickness absence for workers with back pain. Cochrane Database Syst Rev 8: CD001822.
17. Norlund A, Ropponen A, Alexanderson K (2009) Multidisciplinary interventions: review of studies of return to work after rehabilitation for low back pain. J Rehabil Med 41: 115-121.
18. Ballabeni P, Burrus C, Luthi F, Gobelet C, Deriaz O (2011) The effect of recalled previous work environment on return to work after a rehabilitation program including vocational aspects for trauma patients. J Occup Rehabil 21: 43-53.
19. Iakova M, Ballabeni P, Erhart P, Seichert N, Luthi F, et al. (2012) Self perceptions as predictors for return to work 2 years after rehabilitation in orthopedic trauma inpatients. J Occup Rehabil 22: 532-540.
20. Luthi F, Stiefel F, Gobelet C, Rivier G, Deriaz O (2011) Rehabilitation outcomes for orthopaedic trauma individuals as measured by the INTERMED. Disabil Rehabil 33: 2544-2552.

21. Hilfiker R, Bachmann LM, Heitz CA, Lorenz T, Joronen H, et al. (2007) Value of predictive instruments to determine persisting restriction of function in patients with subacute non-specific low back pain. Systematic review. Eur Spine J 16: 1755-1775.
22. Heitz CA, Hilfiker R, Bachmann LM, Joronen H, Lorenz T, et al. (2009) Comparison of risk factors predicting return to work between patients with subacute and chronic non-specific low back pain: systematic review. Eur Spine J 18: 1829-1835.
23. Clay FJ, Fitzharris M, Kerr E, McClure RJ, Watson WL (2012) The association of social functioning, social relationships and the receipt of compensation with time to return to work following unintentional injuries to Victorian workers. J Occup Rehabil 22: 363-375.
24. Clay FJ, Newstead SV, McClure RJ (2010) A systematic review of early prognostic factors for return to work following acute orthopaedic trauma. Injury 41: 787-803.
25. Clay FJ, Newstead SV, Watson WL, McClure RJ (2010) Determinants of return to work following non life threatening acute orthopaedic trauma: a prospective cohort study. J Rehabil Med 42: 162-169.
26. Clay FJ, Newstead SV, Watson WL, Ozanne-Smith J, Guy J, et al. (2010) Bio-psychosocial determinants of persistent pain 6 months after non-life-threatening acute orthopaedic trauma. J Pain 11: 420-430.
27. Toien K, Skogstad L, Ekeberg O, Myhren H, Schou Bredal I (2012) Prevalence and predictors of return to work in hospitalised trauma patients during the first year after discharge: a prospective cohort study. Injury 43: 1606-1613.
28. Sloots M, Dekker JH, Bartels EA, Geertzen JH, Dekker J (2010) Reasons for drop-out in rehabilitation treatment of native patients and non-native patients with chronic low back pain in the Netherlands: a medical file study. Eur J Phys Rehabil Med 46: 505-510.
29. McCauley LA (2005) Immigrant workers in the United States: recent trends, vulnerable populations, and challenges for occupational health. AAOHN J 53: 313-319.
30. Ronda Perez E, Benavides FG, Levecque K, Love JG, Felt E, et al. (2012) Differences in working conditions and employment arrangements among migrant and non-migrant workers in Europe. Ethn Health 17: 563-577.
31. Burrus C, Ballabeni P, Deriaz O, Gobelet C, Luthi F (2009) Predictors of nonresponse in a questionnaire-based outcome study of vocational rehabilitation patients. Arch Phys Med Rehabil 90: 1499-1505.
32. Luthi F, Deriaz O, Vuistiner P, Burrus C, Hilfiker R (2014) Predicting Non Return to Work after Orthopaedic Trauma: The Wallis Occupational Rehabilitation RisK (WORRK) Model. PLoS One 9: e94268.
33. Clayton S, Bambra C, Gosling R, Povall S, Misso K, et al. (2011) Assembling the evidence jigsaw: insights from a systematic review of UK studies of individual-focused return to work initiatives for disabled and long-term ill people. BMC Public Health 11: 170.
34. Stiefel FC, de Jonge P, Huyse FJ, Guex P, Slaets JP, et al. (1999) "INTERMED": a method to assess health service needs. II. Results on its validity and clinical use. Gen Hosp Psychiatry 21: 49-56.
35. Scerri M, de Goumoens P, Fritsch C, Van Melle G, Stiefel F, et al. (2006) The INTERMED questionnaire for predicting return to work after a multidisciplinary rehabilitation program for chronic low back pain. Joint Bone Spine 73: 736-741.
36. Tan G, Jensen MP, Thornby JI, Shanti BF (2004) Validation of the Brief Pain Inventory for chronic nonmalignant pain. J Pain 5: 133-137.
37. Mayer TG, Barnes D, Kishino ND, Nichols G, Gatchel RJ, et al. (1988) Progressive isoinertial lifting evaluation. I. A standardized protocol and normative database. Spine (Phila Pa 1976) 13: 993-997.
38. Vermeulen SJ, Anema JR, Schellart AJ, Knol DL, van Mechelen W, et al. (2011) A participatory return-to-work intervention for temporary agency workers and unemployed workers sick-listed due to musculoskeletal disorders: results of a randomized controlled trial. J Occup Rehabil 21: 313-324.

39. Johnston V, Strong J, Gargett S, Jull G, Ellis N (2013) Enhancing the vocational outcomes of people with chronic disabilities caused by a musculoskeletal condition: Development and evaluation of content of self-management training modules. Work.
40. Zwerenz R, Gerzymisch K, Edinger J, Holme M, Knickenberg RJ, et al. (2013) Evaluation of an internet-based aftercare program to improve vocational reintegration after inpatient medical rehabilitation: study protocol for a cluster-randomized controlled trial. Trials 14: 26.
41. Gabbe BJ, Harrison JE, Lyons RA, Jolley D (2011) Modelling long term disability following injury: comparison of three approaches for handling multiple injuries. PLoS One 6: e25862.
42. Matsuzaki H, Narisawa H, Miwa H, Toishi S (2009) Predicting functional recovery and return to work after mutilating hand injuries: usefulness of Campbell's Hand Injury Severity Score. J Hand Surg Am 34: 880-885.
43. MacKenzie EJ, Bosse MJ, Kellam JF, Pollak AN, Webb LX, et al. (2006) Early predictors of long-term work disability after major limb trauma. J Trauma 61: 688-694.
44. Committee on Injury Scaling (1998) The Abbreviated Injury Scale (AIS-98). Des Plaines, IL, USA: Association for the advancement of automotive medicine.
45. Dworkin RH, Turk DC, Wyrwich KW, Beaton D, Cleeland CS, et al. (2008) Interpreting the clinical importance of treatment outcomes in chronic pain clinical trials: IMMPACT recommendations. J Pain 9: 105-121.
46. Vlaeyen JW, Kole-Snijders AM, Boeren RG, van Eek H (1995) Fear of movement/(re)injury in chronic low back pain and its relation to behavioral performance. Pain 62: 363-372.
47. Osman A, Barrios FX, Kopper BA, Hauptmann W, Jones J, et al. (1997) Factor structure, reliability, and validity of the Pain Catastrophizing Scale. J Behav Med 20: 589-605.
48. Bjelland I, Dahl AA, Haug TT, Neckelmann D (2002) The validity of the Hospital Anxiety and Depression Scale. An updated literature review. J Psychosom Res 52: 69-77.
49. Borloz S, Trippolini MA, Ballabeni P, Luthi F, Deriaz O (2012) Cross-cultural adaptation, reliability, internal consistency and validation of the Spinal Function Sort (SFS) for French- and German-speaking patients with back complaints. J Occup Rehabil 22: 387-393.
50. Konzelmann M, Burrus C, Hilfiker R, Rivier G, Deriaz O, et al. (2014) Cross-Cultural Adaptation, Reliability, Internal Consistency and Validation of the Hand Function Sort (HFS(c)) for French Speaking Patients with Upper Limb Complaints. J Occup Rehabil.
51. Andersson EI, Lin CC, Smeets RJ (2010) Performance tests in people with chronic low back pain: responsiveness and minimal clinically important change. Spine (Phila Pa 1976) 35: E1559-1563.
52. Peters MJ, van Nes SI, Vanhoutte EK, Bakkers M, van Doorn PA, et al. (2011) Revised normative values for grip strength with the Jamar dynamometer. J Peripher Nerv Syst 16: 47-50.
53. Hulley SB, Cummings SR, Browner WS, Grady DG, Newman TB (2013) Designing clinical research. 367 p.
54. Monnin D, Perneger TV (2002) Scale to measure patient satisfaction with physical therapy. Phys Ther 82: 682-691.
55. Steyerberg EW, Moons KG, van der Windt DA, Hayden JA, Perel P, et al. (2013) Prognosis Research Strategy (PROGRESS) 3: prognostic model research. PLoS Med 10: e1001381.
56. Hingorani AD, Windt DA, Riley RD, Abrams K, Moons KG, et al. (2013) Prognosis research strategy (PROGRESS) 4: stratified medicine research. BMJ 346: e5793.
